# Complexity and Challenges of the Clinical Diagnosis and Management of Long COVID

**DOI:** 10.1001/jamanetworkopen.2022.40332

**Published:** 2022-11-03

**Authors:** Ann M. O’Hare, Elizabeth K. Vig, Theodore J. Iwashyna, Alexandra Fox, Janelle S. Taylor, Elizabeth M. Viglianti, Catherine R. Butler, Kelly C. Vranas, Mark Helfand, Anaïs Tuepker, Shannon M. Nugent, Kara A. Winchell, Ryan J. Laundry, C. Barrett Bowling, Denise M. Hynes, Matthew L. Maciejewski, Amy S. B. Bohnert, Emily R. Locke, Edward J. Boyko, George N. Ioannou

**Affiliations:** 1 Health Services Research & Development Center of Innovation for Veteran-Centered and Value-Driven Care, VA Puget Sound Health Care System, Seattle, Washington; 2 Hospital and Specialty Medicine and Geriatrics and Extended Care Services, VA Puget Sound Health Care System, Seattle, Washington; 3 Department of Medicine, University of Washington, Seattle; 4 Pulmonary and Critical Care Medicine, Department of Health Policy & Management, School of Public Health, Johns Hopkins University, Baltimore, Maryland; 5 Seattle Epidemiologic Research and Information Center, VA Puget Sound, Seattle, Washington; 6 Department of Anthropology, University of Toronto, Toronto, Canada; 7 Department of Internal Medicine Division of Pulmonary and Critical Care Medicine, University of Michigan, Ann Arbor; 8 Center to Improve Veteran Involvement in Care, VA Portland Health Care System, Portland, Oregon; 9 Oregon Health & Science University, Portland; 10 Geriatric Research Education and Clinical Center, Durham VA Medical Center, Durham, North Carolina; 11 Department of Medicine, Duke University, Durham, North Carolina; 12 College of Public Health and Human Sciences and Center for Quantitative Life Sciences, Oregon State University, Corvallis; 13 Center of Innovation to Accelerate Discovery and Practice Transformation, Durham VA Medical Center, Durham, North Carolina; 14 Department of Population Health Sciences, Duke University, Durham, North Carolina; 15 Division of General Internal Medicine, Department of Medicine, Duke University, Durham, North Carolina; 16 VA Center for Clinical Management Research, Ann Arbor VA, Ann Arbor, Michigan; 17 Departments of Anesthesiology and Psychiatry, University of Michigan Medical School, Ann Arbor

## Abstract

**Importance:**

There is increasing recognition of the long-term health effects of SARS-CoV-2 infection (sometimes called *long COVID*). However, little is yet known about the clinical diagnosis and management of long COVID within health systems.

**Objective:**

To describe dominant themes pertaining to the clinical diagnosis and management of long COVID in the electronic health records (EHRs) of patients with a diagnostic code for this condition (*International Statistical Classification of Diseases and Related Health Problems, Tenth Revision* [*ICD-10*] code U09.9).

**Design, Setting, and Participants:**

This qualitative analysis used data from EHRs of a national random sample of 200 patients receiving care in the Department of Veterans Affairs (VA) with documentation of a positive result on a polymerase chain reaction (PCR) test for SARS-CoV-2 between February 27, 2020, and December 31, 2021, and an *ICD-10* diagnostic code for long COVID between October 1, 2021, when the code was implemented, and March 1, 2022. Data were analyzed from February 5 to May 31, 2022.

**Main Outcomes and Measures:**

A text word search and qualitative analysis of patients’ VA-wide EHRs was performed to identify dominant themes pertaining to the clinical diagnosis and management of long COVID.

**Results:**

In this qualitative analysis of documentation in the VA-wide EHR, the mean (SD) age of the 200 sampled patients at the time of their first positive PCR test result for SARS-CoV-2 in VA records was 60 (14.5) years. The sample included 173 (86.5%) men; 45 individuals (22.5%) were identified as Black and 136 individuals (68.0%) were identified as White. In qualitative analysis of documentation pertaining to long COVID in patients’ EHRs 2 dominant themes were identified: (1) clinical uncertainty, in that it was often unclear whether particular symptoms could be attributed to long COVID, given the medical complexity and functional limitations of many patients and absence of specific markers for this condition, which could lead to ongoing monitoring, diagnostic testing, and specialist referral; and (2) care fragmentation, describing how post–COVID-19 care processes were often siloed from and poorly coordinated with other aspects of care and could be burdensome to patients.

**Conclusions and Relevance:**

This qualitative study of documentation in the VA EHR highlights the complexity of diagnosing long COVID in clinical settings and the challenges of caring for patients who have or are suspected of having this condition.

## Key Points

**Question:**

What themes pertaining to long COVID can be identified in qualitative analysis of health records from the Department of Veterans Affairs health system?

**Findings:**

This qualitative study including health records from 200 randomly sampled veterans identified 2 dominant themes: (1) clinical uncertainty: it was often unclear whether particular symptoms were due to long COVID, given the medical complexity and functional limitations of many patients and absence of specific markers for this condition, which led to ongoing monitoring, diagnostic testing, and referral; and (2) care fragmentation: post–COVID-19 care processes were often siloed from other care and could be burdensome to patients.

**Meaning:**

These findings highlight the complexity of diagnosing and managing long COVID in clinical settings.

## Teaser

This qualitative study describes dominant themes pertaining to the clinical diagnosis and management of long COVID in the electronic health records of veterans with a diagnostic code for this condition.

## Introduction

There is increasing interest in long COVID, defined in December 2021 by the World Health Organization (WHO) as “the illness that occurs in people who have a history of probable or confirmed SARS-CoV-2 infection; usually within three months from the onset of COVID-19, with symptoms and effects that last for at least two months. The symptoms and effects… cannot be explained by an alternative diagnosis.”^1^ Although a variety of other definitions of long COVID have been proposed, all include a wide range of different signs and symptoms, including fatigue, dyspnea, and cardiovascular, cognitive, mental health, olfactory, and gustatory symptoms that emerge or persist well beyond the period of initial infection with SARS-CoV-2.^2^

Estimates of the incidence of long COVID vary widely depending on the specific definition used, the population studied, the source data, and the duration of follow-up.^3,4,5,6,7,8,9,10^ Nonetheless, lower bound estimates suggest that at least 12% of individuals initially infected with SARS-CoV-2, or approximately 9.6 million people currently living in the US, may have developed long COVID.^11,12^

Individuals who themselves have experienced long COVID have played a pivotal role in raising public and professional awareness of this condition by sharing their first-hand accounts in the scientific literature and lay press and through online social media communities.^13,14,15^ Indeed, it is perhaps a testament to the sophistication and effectiveness of the long COVID advocacy community^14^ that the patient-originated term “long COVID” has now entered the medical lexicon^14,15^ and that efforts are underway by medical professionals and health systems to better address the needs of those affected.^16^

Qualitative studies^17,18,19,20,21,22,23^ have offered rich descriptions of the experiences and perspectives of individuals with long COVID, and epidemiologic studies^8,9,24,25^ describing the incidence of long COVID are beginning to appear in the literature. However, to our knowledge, no prior studies have offered a detailed description of the clinical diagnosis and management of long COVID within health care systems. To address this knowledge gap, we conducted a qualitative analysis of the electronic health records (EHRs)^26,27,28,29^ of a national random sample of patients with a long COVID diagnostic code^6^ receiving care in the Department of Veterans Affairs (VA). Our specific research goal was to understand how clinicians approached the diagnosis of long COVID and the challenges they faced in making this diagnosis and caring for patients who had or were suspected to have long COVID.

## Methods

This qualitative study was approved by the VA Puget Sound Health Care System institutional review board, which waived the requirement for informed consent because risks associated with medical record review are minimal. Our study was conducted in accordance with relevant portions of the Consolidated Criteria for Reporting Qualitative Research (COREQ) reporting guideline.

### Cohort Assembly

To assemble our cohort, we used the VA COVID-19 Shared Data Resource (CSDR),^30^ VA’s comprehensive COVID-19 repository. We first constructed a cohort of all veterans with documentation of at least 1 positive polymerase chain reaction (PCR) test result for SARS-CoV-2 recorded in VA data sources between February 27, 2020, and December 31, 2021, and at least 1 primary care visit within the VA during the preceding 18-month period. A total of 260 692 veterans met these criteria. To focus our medical record review on cohort members most likely to have experienced long COVID, we identified a subset of 4338 cohort members with at least 1 entry of the novel *International Statistical Classification of Diseases and Related Health Problems, Tenth Revision* (*ICD-10*) diagnostic code for long COVID (U09.9) in VA data sources between October 1, 2021, when the code was implemented,^6^ and March 1, 2022. We then conducted a detailed review of the EHRs of a national random sample of 200 of these patients. Information on sampled patients’ demographic and clinical characteristics at the time of their first positive PCR test result for SARS-CoV-2 in VA records (study baseline) and health care utilization during the subsequent 30-day period were ascertained from the CSDR. Information on race, ethnicity, and sex were ascertained from the CSDR as recorded in the VA Vital Status File and included for descriptive purposes. Comorbid conditions in CSDR are defined based on *ICD-10* and *Current Procedural Terminology* (*CPT*) codes and Natural Language Processing of text in the VA-wide EHR during the 2-year period before each patient’s first recorded positive PCR test result for SARS-CoV-2.

### EHR Search

We used a Lucene-based search tool, (Apache Lucene, version 8.11; Apache Software Foundation) developed by 1 of the coauthors (R.J.L.)^26,27,28^ to search text in cohort members’ VA-wide EHRs stored as text integration utilities (TIU) notes in the VA Corporate Data Warehouse (CDW). To capture documentation pertaining to the clinical diagnosis and management of long COVID we used the search term *COVID* (not case sensitive) and applied a filter to limit capture of notes with boilerplate text (eg, text in templated form letters and administrative notes). To ground our analyses in a detailed understanding of each patient’s post–COVID-19 illness trajectory, our search extended from study baseline through each patient’s most recent VA encounter at the time the search index was finalized on April 12, 2022. During the observation period, there were 14 203 notes with at least 1 mention of the term *COVID* in the records of the 200 cohort members. After applying the filter, our search identified 7708 notes with at least 1 mention of this term in the records of 198 cohort members (median [IQR] 21.5 [9-50] notes per patient). We intentionally did not limit our search to more specific terms related to long COVID (eg, *long COVID*, *long haul*, *post-acute sequelae of COVID-19*, *PASC*), as these were not commonly documented in patients’ EHRs during the observation period. One of the coauthors (A.M.O., a VA nephrologist and physician scientist with qualitative research experience) then reviewed the content of all notes captured in our search, with a particular focus on text surrounding *COVID* mentions. In rare instances where the clinical context of particular mentions of the term *COVID* was not clear after reviewing the specific note captured in our search, A.M.O. reviewed surrounding notes in patients’ EHRs not captured in our search as needed to understand the clinical context.

### Qualitative Analysis

We used inductive content analysis,^31,32,33^ a systematic approach to describing phenomena with the goal of providing novel insights and helping to generate hypotheses, to analyze text in patients’ EHRs pertaining to the clinical diagnosis and management of long COVID. Using the search tool, 1 coauthor (A.M.O.) reviewed, abstracted, and coded all potentially informative text in notes captured in our search using the constant comparative method,^34,35^ developing an initial list of themes and subthemes and identifying a shortlist of candidate exemplar quotations. A second coauthor (E.K.V., a geriatrician and palliative care physician with qualitative research experience), independently coded a subset of abstracted phrases, accessing the EHR as needed to clarify the clinical context of abstracted phrases. The 2 coauthors then worked collaboratively to reach consensus on emerging themes and subthemes, resolve any differences in interpretation, and develop and refine the thematic descriptions and schema, selecting exemplar quotations for inclusion in draft manuscript tables. Coauthors with experience in qualitative research (T.J.I., J.S.T., E.M.V., C.R.B., K.C.V., M.H., A.T., S.M.N., K.A.W., C.B.B., and E.R.L.) from a variety of different disciplines and specialties provided detailed input on thematic organization and alignment between exemplar quotations and thematic descriptions. During this iterative process, A.M.O. and E.K.V. returned to abstracted phrases and the EHR as needed to elucidate reasons for differences in interpretation, clarify meaning, confirm that selected themes were grounded in the data, and review the choice of exemplar quotations. This approach of incorporating input from team members with differing exposure to the source data seeks to balance the need for immersive EHR review—which is time-consuming and requires both expertise in qualitative research and an intimate understanding of VA clinical care and related documentation processes—with the need to incorporate alternative viewpoints and interpretations, build consensus around the content and organization of thematic descriptions, and establish trustworthiness.^26,27,28,29^ Data were analyzed from February 5 to May 31, 2022.

## Results

### Cohort Characteristics

At study baseline, the mean (SD) age of the 200 sampled patients was 60 (14.5) years, including 92 patients (46.0%) aged younger than 60 years, 75 patients (37.5%) aged 60 to 74 years, and 43 patients (16.5%) aged 75 years or older (Table 1). There were 173 men (86.5%); 45 patients (22.5%) were identified as Black in the CSDR, 136 patients (68.0%) were identified as White, 6 patients (3.0%) were identified as members of other racial groups (eg, American Indian or Alaskan Native, Asian, Native Hawaiian or other Pacific Islander), and 13 patients (6.5%) were missing information on race. Overall, 63 patients (31.5%) had diabetes, 54 patients (27.0%) had chronic obstructive pulmonary disease, 16 patients (8.0%) had heart failure, 85 patients (42.5%) had major depressive disorder, and 54 patients (27.0%) had posttraumatic stress disorder (Table 1). Overall, 60 patients (30.0%) were hospitalized and 8 patients (4.0%) received mechanical ventilation at a VA facility within 30 days of their first positive test result for SARS-CoV-2 recorded in VA sources. Patients from all VA regional service networks were represented in the sample, including 12 patients (6.0%) who were infected before May 31, 2020; 28 patients (14.0%) who were infected between June 1 and October 31, 2020; 67 patients (33.5%) who were infected between November 1, 2020, and April 30, 2021; 41 patients (20.5%) who were infected between May 1, and September 30, 2021; and 52 patients (26.0%) who were infected between October 1 and December 31, 2021. The median (IQR) time from study baseline to the earliest documentation of a diagnostic code for long COVID was 287 (48-385) days (Table 1).

**Table 1.** Baseline Characteristics of the 200 Sampled Patients With a Diagnostic Code for Long COVID

Characteristics	No. (%)
Age, y	
Mean (SD)	60.0 (14.5)
	
<60	92 (46.0)
60-74	75 (37.5)
≥75	43 (16.5)
Sex	
Men	173 (86.5)
Women	27 (13.5)
Race	
Black	45 (22.5)
White	136 (68.0)
Other^a^	6 (3.0)
Missing	13 (6.5)
Comorbidities	
Diabetes	63 (31.5)
Congestive heart failure	16 (8.0)
Chronic obstructive pulmonary disease	54 (27.0)
Posttraumatic stress disorder	54 (27.0)
Major depressive disorder	85 (42.5)
Charlson Comorbidity index, median (IQR)	1 (0-3)
Care during initial infection	
Hospitalized within 30 d	60 (30.0)
Mechanical ventilation within 30 d	8 (4.0)
Time period of first positive PCR test result for SARS-CoV-2	
Before May 20, 2020	12 (6.0)
June 1-October 31, 2020	28 (14.0)
November 1, 2020, to April 30, 2021	67 (33.5)
May 1-September 30, 2021	41 (20.5)
October 1-December 31, 2021	52 (26.0)
Time from baseline to earliest diagnostic code for long COVID, median (IQR), d	287 (48-385)

Abbreviation: PCR, polymerase chain reaction.

^a^ Other race includes American Indian or Alaskan Native, Asian, Native Hawaiian or other Pacific Islander.

### Thematic Analysis

We identified 2 dominant themes pertaining to the diagnosis and management of long COVID based on documentation in the EHRs of veterans with a diagnostic code for this condition. The first theme was clinical uncertainty (Table 2), and the second was care fragmentation (Table 3).

**Table 2.** Theme 1: Clinical Uncertainty

Quotation No.	Note title	Date	Exemplar quotation^a^
**Encountering medical complexity**			
1	Primary care note	January 2022	“This is a 56 [year-old] MALE who presents for…evaluation of ongoing [shortness of breath] and dizziness, post-COVID—[symptom] onset [13 mo prior], confirmed positive. Never had these [symptoms] in the past, only began with COVID [diagnosis]. Pt notes dyspnea worse while doing housework, and states he tried to walk 1-block, and had to stop multiple times–previously very active, walking >20k steps per day at work.…Associated generalized weakness–feels like I struggle to open bottles and pick up heavy load of laundry, etc. Associated dizziness described as ‘room spinning’ after extensive movement, resolves spontaneously in 1-2 minutes after sitting down.”
2	Emergency department note	November 2021	“Patient comes in with worsening symptoms after recent Covid about a month ago has completed a course of steroids for that. Will treat him as a bronchitis with steroids and breathing treatments got a scan to make sure there was no signs of blood clot or structural abnormalities and will admit for further care.”
3	Primary care note	June 2021	“Post-Covid Syndrome: - fatigue/[dyspnea on exertion], loss of taste...and brain foggy-advised on general supportive care and moderation in exercise/activity.”
4	Pulmonary consult	May 2021	“She also relates a history of asthma and chronic cough. Difficult at this time to separate out post COVID recovery from potential underlying asthma.”
5	Social work note	August 2021	“[Patient] has several different cognitive issues that affect his memory and ability to process frustrations. He has [traumatic brain injury] from a self-inflicted gunshot wound to his head, the aftermath of long-term COVID-19, and the effects of long-term alcoholism.”
6	Mental health outpatient note	May 2021	“Veteran with persistent, severe anxiety that has been exacerbated by long COVID complications that have him on short-term disability from work. Reports secondary depression symptoms and is very interested in starting therapy for anxiety and depression.”
7	Physician emergency department note	March 2021	“She [patient’s wife] expressed concerns about [patient’s] ongoing substance abuse and deterioration of cognitive functioning ever since he was on ventilator due to complications of COVID-19 infection.”
8	Mental health note	January 2021	“Veteran reported primary concerns is panic attacks, increasing in frequency/intensity over the past 3+ months in the context of various psychosocial stressors (COVID Pandemic, 4 children at home, working part time, planning/getting married…moving…contracting COVID).”
9	Geriatrics and extended care note	August 2020	“Veteran states he has been ‘feeling pretty good.’ He states his sense of smell has almost recovered [after COVID-19], rating it at 9/10; denies issues with sense of taste. He states he has been getting daily morning headaches, but also admits to not wearing his [continuous positive airway pressure device] over the last couple of days.”
**Limited functional reserve**			
10	Nursing treatment plan note	June 2020	“This is a 70 [year-old] Resident, now prior positive Covid 19.…Resident continues to be limited to extensive assist with his [activities of daily living] however due to generalized weakness [due to] COVID 19, he tires very easily and does not have as much motivation to leave the room. Prior to COVID, Resident would walk an average of 400 ft using walker with supervision.…He is weaker, especially in lower extremities, which may have contributed to his most recent fall.”
11	Attending H&P note	April 2021	“82 [year-old] male veteran was brought to the emergency room by his wife with complaints of generalized weakness since several weeks, right lower extremity weakness since yesterday. The patient had a severe COVID-19 infection [3 mo prior], since then he has been hospitalized at least 7 times according to his wife. He had a 50-pound weight loss, decreased appetite and was taken off metformin, glipizide due to hypoglycemia.”
12	Physical therapy consult note	January 2021	“[Patient] is a 77 [year-old] male who was admitted…via the [emergency department] secondary to [shortness of breath]/fatigue, [status post] COVID [pneumonia] [approximately] 6 weeks ago. [Patient] with multiple hospitalizations and subsequent [rehabilitation] stay, however, still with compromised respiratory state preventing [patient] from safely and reliably functioning in a home environment.”
**Reliance on patient reports**			
13	Geriatrics and extended care note	July 2021	“Patient shares she is doing much better. Her fatigue has improved over 50%. She is no longer fatigued all day long. Now she is able [to] work and feel[s] almost her normal baseline fatigue from working night shift.”
14	Neurology note	March 2021	“55 [year-old] woman with history of…migraines, obesity who presents with headache and recent worsening of headaches since COVID infection [3 mo prior]. Duration of pain is constant, and not [episodic] like her prior migraine headaches. She is rarely headache free.”
15	Psychology note	March 2022	“‘I still suffer with a little breathing problem at different times my [girlfriend] helps me with getting dressed, I leave early in the AM, she lays out my stuff, to help me not have to move around a lot, she helps me [with] things that I have to…bend [for].’”
16	Mental health telephone note	January 2022	“Veteran goes on to talk about ongoing pain which he describes as ’whole body pain,’ difficulty breathing, and headaches. He associates his increase in physical chronic pain to his [diagnosis] of Covid in the summer of 2020; however, a review of past [mental health] notes indicate that he has consistently been complaining of somatic issues similar to today for 10+ years.”
**Monitoring, diagnostic testing, and referral**			
17	Cardiology outpatient note	March 2021	“I see that the [patient’s hemoglobin] has improved and [platelet] count has normalized; I would not intervene at this point. His [complete blood count] abnormalities are related to his COVID19 infection where a severe acute phase response (causing primarily thrombocytopenia) turns chronic and [patients] are as a result susceptible to normocytic anemia. Given the trend, I would simply follow over time.”
18	Primary care letters	February 2021	“The COVID pneumonia is still evident in your lungs as expected. This will take some time/months to resolve, however it is getting better slowly. The spots/nodules in your lungs are all stable/no change. The fluid in your lung has resolved. We will repeat the chest CT in 6 months and repeat a chest x-ray next month to follow up on the pneumonia.”
19	Pulmonary note	September 2021	“His ongoing dyspnea after his [13 mo prior] COVID19 infection appears to be more so due to deconditioning since he admits to not being as active vs concern for long-COVID or post COVID 19 fibrosis. Will plan to order a Chest CT to help evaluate.”
20	Letters	September 2021	“Dear [patient]: It was good to see you at your recent COVID Aftercare clinic appointment. Based on our evaluations we recommend the following: We are ordering a cardiac evaluation to make sure heart issues are not causing/contributing to your fatigue and activity intolerance.”
21	Primary care telephone encounter	January 2021	“Please let [patient] know that his fatigue is most likely related to obstructive sleep apnea, and he may have some residuals from COVID-19. He really needs to be on [positive airway pressure] at night. Please find out if he is agreeable to Sleep Disorders referral to get started on [positive airway pressure].”
22	Geriatric medicine consult	December 2021	“May have [mild cognitive impairment] that could have begun prior to his COVID-19 infection, [Montreal Cognitive Assessment Test] score appropriate given his age and demographics however [patient] would likely benefit from continued testing and follow up with Psychology.”
23	Letters	July 2021	“Below are the results from your recent pulmonary testing for the VA Post-COVID-19 Convalescence Program. [pulmonary function tests] (breathing tests), [date]: normal spirometry, mildly reduced total lung capacity, and moderately reduced diffusion capacity. [6-minute walk test] (walking test): not done. CT chest scan, [date]: significantly improved since prior CT in [January 2020], but there are mild residual post-Covid-19 pneumonia changes. Recommendations: 1. Repeat breathing tests…now since it has already been 6-months since your acute Covid-19 pneumonia. 2. Repeat CT chest scan in 3 months to closely monitor the residual changes after your Covid-19 pneumonia.”
24	Telehealth note	November 21	“While I feel her fatigue is very much likely due to her anemia, will re-order echo to help rule out cardiac issues contributing to her fatigue.…Not unreasonable given history of COVID.…Will also place allergy consult as patient wishes to have a second opinion regarding treatment of her hives. Despite having fatigue, patient is at her functional baseline.…At this time there is no need for further follow up in the post COVID virtual clinic.”

Abbreviations: CT, computed tomography; echo, echocardiogram; H&P, history and physical examination; k, thousand; Pt, patient; VA, Department of Veterans Affairs; x-ray, radiograph.

^a^ Square backets include text that is altered from the original to spell out medical acronyms and abbreviations and clarify meaning.

**Table 3.** Theme 2: Care Fragmentation

Quotation No.	Note title, signatory	Date	Exemplar quotation
**Siloed approach**			
25	Pulmonary telephone encounter note	May 2021	“Notified by…[registered nurse] that the [patient] was calling for CT chest results ordered by the Covid Convalescent Clinic…I called and spoke with [patient] regarding the CT chest results. I let him know he had residual findings from his Covid infection and a couple of very small nodules. I let him know I did not know the routine from the Covid Convalescent Clinic and he would have to wait to hear from them again as far as a plan goes…Plan: 1. Follow up with the Covid Convalescent Clinic for plan regarding CT chest results.”
26	Geriatrics and extended care note (COVID convalescences telephone follow-up note)	August 2021	“COVID Course: COVID + [13 mo prior] with associated symptoms of [shortness of breath] and fever. [admission] [approximately 9 wk long] for COVID [pneumonia] with acute hypoxic respiratory failure with superimposed influenza and given dexamethasone; declined remdesivir/convalescent plasma. During prolonged hospitalization: [right ventricular] intracardiac thrombi, [acute kidney injury] secondary to [vancomycin] toxicity, disseminated candidemia, [gastrointestinal bleed] bleed…fungemia with endopthalmitis, aspergillosis and [gastrostomy] tube placement…intubated [date], [tracheostomy] [date], [tracheostomy] collar [date]. Discharged to [long-term acute care hospital]…reinfected with COVID again [8 mo prior].”
27	Sleep medicine consult	July 2021	“Not able to use [positive airway pressure] therapy at this time. He had COVID in [10 mo prior] (hospitalized), discharged on oxygen which was discontinued in March. He developed Shingles (on face) and subsequently, now undergoing treatment for skin cancer on face and scalp.”
28	Hematology and oncology consult	February 2021	“[Patient] is a 77 [year-old] man with history of [chronic lymphocytic leukemia]. Clinically, he stated that he is feeling well. He spent 2-3 hours off [oxygen] every day and walking. He has no bleeding. He had been in and out of the hospital with COVID…he has no fever, chills but still has some night sweat since covid, but getting better. He bruised easy and has some [shortness of breath] once a while.”
29	Physician outpatient note	June 2021	“Assessment/plan: 3. Chronic fatigue, [status post] COVID infection. Regular exercise as tolerated. 4. Sleep difficulty (sleep study results pending). [follow-up] with Sleep clinic.”
**Lack of care coordination**			
30	Geriatrics and extended care note	May 2021	“Spoke to veteran who stated that he was in the process of applying for an extension with his [state disability] through his work. He reports that his [primary care practitioner] has sent some information but when he talked to the [human resources] representative on Friday they were requesting clinical notes. Veteran reports he has been printing summaries from his [patient portal] and sending over notes and diagnostics to them already. He states he thinks his [primary care] team is fed up with him so that’s why he decided to outreach to convalescence. Advised that per [physician’s] note, [primary care practitioners] are usually the ones tasked to complete disability paperwork. Veteran reports he needs to submit the paperwork by tomorrow because they will have a meeting on Wednesday to determine if they will approve or deny the extension.…Nurse provided assistance to veteran.”
31	Primary care secure messaging	April 2021	“I was unaware you had the echocardiogram, it was ordered by the pulmonologist you saw to discuss after effects of COVID. Good news is that it looked pretty good. Your x-ray also looked fine. I will let [pulmonologist] know to reach out to you to discuss further.”
32	Telephone encounter note	September 2021	“[Patient] SAYS HE HAD A CT DONE WHICH WAS ORDERED BY THE COVID TEAM. HE HAS NOT RECEIVED ANY RESULTS….CT SHOWS L UPPER LOBE [pulmonary] NODULE WHICH NO ONE TOLD [patient] ABOUT, MY TELLING HIM IS NEW INFORMATION TO HIM.”
33	Psychology note	January 2022	“This 52 [year-old], Hispanic, male veteran was referred by treating provider with a request to assess psychological functioning related to past [diagnosis] of COVID-19.…During follow-up with a Convalescence provider he reported increase in tinnitus symptoms, hair loss, fatigue, forgetfulness and more easily distracted. During this evaluation a slight increase in brain fog, pain, and depression symptoms since his COVID illness. He has been followed by audiology recently due to his increased tinnitus. He has also recently been seen in the post-COVID [neurorehabilitation] program for evaluation.…He denied a need for additional [mental health] services at this time. His cognitive screening assessment was within normal limits and does not indicate a need for further neuropsychological assessment.”
34	Pulmonary telephone encounter	December 2021	“72 [year-old] previously seen purely because his community care pulmonary visit was delayed. [Patient] with chronic hypoxic [respiratory] failure from long COVID, restrictive lung disease [interstitial lung disease] (likely COVID related), [right lower lobe] small nodule, [mediastinal lymphadenopathy], [obstructive sleep apnea]. [Patient’s] only pulmonary note from community is from [five months prior]. They sent the same serologies we sent here (all were negative here) and referred him to the specialty [interstitial lung disease] clinic at [non-VA medical center].”
**Potentially burdensome care**			
35	Primary care note	November 2021	“Patient has asked if [primary care practitioner] can consult to see the [non-VA] Post-Covid Clinic. States that he is still experiencing insomnia at night but is fatigued throughout the day. States, ‘I just don't think I got over this thing.’”
36	Care coordination home telehealth note	June 2021	“He likely also has long COVID [symptoms] after his second infection. I offered to request nocturnal pulse oximetry via pulmonology, but he declined for now. He will talk with his cardiologist next week.”
37	Geriatrics and extended care note	January 2022	“Spoke with the patient to discuss the COVID Convalescence Program. Veteran was first [diagnosed] with COVID [6 mo prior]. He reports he recovered fully and has no residual complaints. Screening tool did have a few…indicators such as memory loss, fatigue and changes to breathing. All were very mild and Veteran attributes these to age. He declines enrollment.”
38	Geriatrics and extended care note	May 2021	“Veteran stating that he wants to leave the COVID CONVALESCENCE program because he says ‘I am still monitored by y’all and I cannot leave my house and travel without a nurse calling me.’”
39	Physical medicine rehab consult	February 2022	“98 [year-old male] Veteran…with a history of COVID-19 [3 mo prior] admitted to emergency department from home with [complaint of] right rib pain.…[Patient] admits to having 1-2 falls in the past several months. [Patient] states he has ‘accidents’ when he loses his balance.…He endorses having mild clutter in his home which he states he will ask the [home health agency] to assist with clearing. [Patient] declines need for a stair glide or grab bars. [Patient] states ‘I am 98, I am not going to start putting things in my house now.’”

Abbreviations: CT, computed tomography; L, left; VA, Department of Veterans Affairs; x-ray, radiograph.

^a^ Square backets include text that is altered from the original to spell out medical acronyms and abbreviations and clarify meaning.

#### Clinical Uncertainty

There was often uncertainty about whether particular symptoms were due to long COVID, given the medical complexity and functional limitations of many cohort members and the absence of disease-specific markers for this condition. Uncertainty about the etiology and expected course of patients’ symptoms could prompt ongoing monitoring, diagnostic testing, and specialist referral. Subthemes within the broader theme of clinical uncertainty, with representative quotes, are presented in Table 2.

##### Encountering Medical Complexity

Some cohort members had been fairly healthy prior to contracting SARS-CoV-2 and were able to draw a sharp distinction between their symptoms before and after COVID-19 (quotation 1). Clinicians tended to recognize certain clinical events (eg, pulmonary embolism) (quotation 2) and symptoms (eg, fatigue and cognitive dysfunction) as postacute complications of COVID-19 (quotation 3) (Table 2). However, most patients had 1 or more other comorbid conditions with symptoms that could potentially overlap with those of long COVID. Thus there was often uncertainty about the etiology of potential long COVID symptoms (quotation 4) or recognition that these could be due to a variety of different factors, in addition to COVID-19 (quotation 5) (Table 2). The downstream effects of prior SARS-CoV-2 infection were often seen as interacting in complex ways with patients’ other health conditions (quotation 6) and behaviors (quotation 7), and with a range of situational factors, including socioeconomic stressors related to the pandemic (quotation 8) (Table 2). Changes in the treatments that patients were receiving for their other health conditions added to the difficulty of determining the etiology of particular symptoms (quotation 9) (Table 2).

##### Limited Functional Reserve

Based on our review of documentation in the EHR, many members of our cohort were already functionally impaired at the time of their initial infection with SARS-CoV-2 (quotation 10) (Table 2). For some, infection with SARS-CoV-2 was followed by a series of seemingly unrelated adverse health events (eg, falls, hospital admissions for other causes), with the initial infection functioning as a contributor rather than proximal cause of these events (quotation 11) (Table 2). Some patients had prolonged or repeated hospital admissions or nursing home stays after infection with SARS-CoV-2, blurring the boundaries between the outcomes associated with acute infection, prolonged hospitalization, and long COVID (quotation 12) (Table 2).

##### Reliance on Patient Reports

In the absence of disease-specific markers for long COVID, clinicians relied on patients’ often nuanced personal accounts of how they had been impacted by SARS-CoV-2 (quotation 13) (Table 2). Rather than experiencing an entirely new set of symptoms following infection with SARS-CoV-2, many patients described alterations in the severity or quality of preexisting symptoms (quotation 14) (Table 2). Patients might also describe how COVID-19 had affected their day-to-day functioning and need for help (quotation 15), in ways that might not otherwise have been evident to clinicians (Table 2). While clinicians mostly appeared to accept patients’ accounts of how they had been impacted by COVID-19 at face value, some expressed doubt about patients’ attribution of their symptoms to prior COVID-19 (quotation 16) (Table 2).

##### Monitoring, Diagnostic Testing, and Referral

When faced with uncertainty about the etiology, future course, and optimal management of patients’ underlying symptoms and the possibility that these might be due to long COVID, clinicians sometimes adopted a watchful waiting strategy (quotation 17) (Table 2). However, clinicians more commonly recommended additional testing or specialist referral. We found examples of clinicians obtaining imaging and other tests to monitor for recovery from COVID-19 (quotation 18) (Table 2). Clinicians also routinely recommended additional testing or specialist referral to support or refute the diagnosis of long COVID (quotation 19) and search for alternative potential etiologies of patients’ symptoms (quotations 20 and 21) (Table 2). Diagnostic testing to evaluate post–COVID-19 symptoms could lead to further monitoring, diagnostic testing, and referral (quotation 22), as could enrollment in post–COVID-19 programs (quotations 23 and 24) (Table 2).

#### Care Fragmentation

Post–COVID-19 care processes, including the nascent post–COVID-19 clinics and COVID-19 convalescence telehealth programs that were emerging within and outside the VA during the observation period, were often siloed from and poorly coordinated with other aspects of care and could be burdensome to patients. Subthemes within the broader theme of care fragmentation, with representative quotes, are presented in Table 3.

##### Siloed Approach

Based on documentation in patients’ EHRs, post–COVID-19 care processes seemed to be largely added on to the care that patients were already receiving, with their other clinicians involved only peripherally (quotation 25) (Table 3). Intake for post–COVID-19 programs typically included a detailed account of each patient’s COVID-19 history along with systematic screening for COVID-19–related symptoms and visits with members of a multidisciplinary team (eg, psychology, physical therapy, nutrition. speech and language) (quotation 26) (Table 3). Outside of this context, the notes of patients’ other clinicians often focused on narrow aspects of their post–COVID-19 clinical presentation relevant to the treating discipline or specialty (quotations 27 and 28) (Table 3). Primary care clinic notes tended to list prior SARS-CoV-2 infection as a discrete item in the clinical assessment and plan, disconnected from patients’ other problems and presenting concerns (quotation 29).

##### Limited Care Coordination

Documentation in the EHR suggested that there might be unclear role delineation between the multidisciplinary COVID-19 team and patients’ primary care or other clinicians (quotation 30), requiring a level of collaboration (quotation 31) that did not always happen (quotation 32) (Table 3). Some aspects of post–COVID-19 care might also duplicate care that veterans were already receiving within (quotation 33) or outside (quotation 34) the VA (Table 3).

##### Potentially Burdensome Care

While some veterans were proactive in seeking access to post–COVID-19 care processes (quotation 35), the documentation we reviewed suggested that patients often missed appointments with, or failed to respond to, calls from program staff (Table 3). Patients also sometimes declined some or all aspects of the care that post–COVID-19 programs had to offer (quotations 36 and 37) or found program demands (quotation 38) or clinician recommendations (quotation 39) to be unwelcome or burdensome (Table 3).

## Discussion

The findings of this qualitative analysis of the EHRs of a random national sample of 200 veterans who had experienced SARS-CoV-2 infection and who had at least 1 diagnostic code for long COVID speak to the substantial challenges of diagnosing and managing long COVID in clinical settings. More broadly, they highlight the prominent roles of clinical uncertainty and care fragmentation—familiar themes in US health care—in shaping the care of patients with or suspected of having this condition.

Several reports have suggested that a relatively high percentage of people infected with SARS-CoV-2 will develop long COVID.^6,8^ Our findings among a sample of patients with a diagnostic code for this condition in administrative data highlight the interpretative complexity of identifying and characterizing this emerging syndrome in clinical context. In particular, our findings suggest that the WHO definition of long COVID, which is a diagnosis of exclusion that includes a long list of relatively common and nonspecific symptoms, may prove difficult to operationalize in clinical settings.^36^

Similar to other clinical syndromes with few observable elements of pathology and for which definitive biologic markers are lacking, such as fibromyalgia,^37^ myalgic encephalomyelitis/chronic fatigue syndrome,^38,39,40^ chronic pain,^41^ and Gulf War illness,^42,43^ qualitative studies conducted among patients with long COVID have described the difficulties they face obtaining needed support from health systems and health care professionals.^17,18,19,20,21,22,23^ In contrast, the documentation we reviewed suggested that, at least for this sample of patients with a diagnostic code for long COVID, clinicians were trying to make sense of patients’ symptoms in the context of prior COVID-19 but found this to be fraught with uncertainty. Although clinicians tended to recognize certain health events (eg, pulmonary embolism) to be the direct result of prior infection with SARS-CoV-2, distinguishing the downstream postacute effects of viral infection from overlapping signs and symptoms caused by other health conditions usually was not straightforward and often led to additional testing and specialist referral.^36^

Qualitative studies of the lived experiences of people with long COVID have largely focused on younger individuals recruited through social media channels.^17,18,19,20,21,22,23^ Many of these individuals were relatively healthy prior to SARS-CoV-2 infection, and were able to draw sharp contrasts between their functional status and quality of life before and after contracting SARS-CoV-2.^17,18,19,20,21,22,23^ More recent data suggest that new and persistent symptoms after infection with SARS-CoV-2 are especially common at older ages.^8,9,44,45^ Based on our qualitative analysis of the EHRs of patients with a diagnostic code for long COVID—many of whom were older and had 1 or more underlying health conditions and/or functional limitations—it was often difficult, if not impossible, to disentangle the effects of long COVID from those of other health conditions and related treatments, and from a range of situational factors.^46^ Many members of our cohort experienced not so much the onset of a brand new set of symptoms, but worsening or alterations in the quality of preexisting symptoms. Rather than serving as the proximate cause of adverse health events, prior infection with SARS-CoV-2 was often one among many potential contributing factors.

In this context, the addition of monitoring, diagnostic testing, and specialist referral—often undertaken to exclude other potential etiologies of long COVID symptoms—to the care that patients were already receiving could lead to fragmented and potentially burdensome care. More broadly, our findings highlighting clinical uncertainty and care fragmentation as dominant themes in the treatment and management of patients with or suspected of having long COVID offer an illustration of the potential pitfalls of applying a disease-based approach to the care of older adults with complex medical histories and limited functional reserve.^47^ These findings also highlight the potential value of a more integrative person-centered approach to caring for individuals who have or are suspected of having long COVID.

### Limitations

This study has some limitations. Our results, derived from the integrated VA health care system, might not be transferable to other health systems or patient populations, particularly fee-for-service and for-profit health systems and those with a greater representation of women. The degree to which the newly introduced diagnostic code for long COVID identifies individuals with this condition is not known, and may be changing over time, perhaps limiting the transferability of our findings to more recent time periods and to veterans with long COVID who did not have a diagnostic code for this condition. Additionally, documentation in the EHR provides, at best, an incomplete and indirect understanding of the perspectives and lived experiences of veterans with long COVID and clinicians involved in their care. Nonetheless, analysis of documents can complement the findings of studies based on interpersonal interviews and fieldwork observations by identifying system-level constructs that might not otherwise be identified.^48,49^

## Conclusions

The findings of this qualitative study conducted among a national random sample of veterans who had experienced SARS-CoV-2 infection and who had at least 1 diagnostic code for long COVID in VA administrative data underscore the interpretative complexity of characterizing long COVID in clinical settings. Our findings speak to the challenges of caring for patients who have or are suspected of having long COVID, particularly those who are medically complex and functionally impaired. They also highlight the prominent roles of clinical uncertainty and care fragmentation in shaping the care of members of this population, underscoring the need for a more person-centered and integrative approach.
